# Severe lymphocytopenia induced by methimazole tablets: A rare case report

**DOI:** 10.1097/MD.0000000000045322

**Published:** 2025-11-07

**Authors:** Yi Li, Bo Tan, Liutao Zheng

**Affiliations:** a Department of Pharmacy, Pengzhou Hospital of Traditional Chinese Medicine, Chengdu, Sichuan, China; b Department of Pharmacy, Pengzhou People’s Hospital, Chengdu, China; c Department of Endocrinology, Hospital of Chengdu University of Traditional Chinese Medicine, Chengdu, Sichuan, China.

**Keywords:** adverse drug reactions, lymphocytopenia, methimazole, susceptibility factors

## Abstract

**Rationale:**

Severe lymphocytopenia, febrile granulocytopenia, and leukopenia induced by methimazole (MMI) are extremely rare.

**Patient concerns:**

A 26-year-old Han Chinese female with hyperthyroidism was admitted to the hospital with fever and tonsillar enlargement after taking MMI tablets for 1 month. Adverse drug reactions correlation analysis revealed this as a novel severe adverse reaction.

**Diagnoses:**

The patient was diagnosed with agranulocytosis with fever, thyroid storm, acute suppurative tonsillitis, and mild anemia.

**Interventions:**

After admission, MMI tablets were discontinued. The patient received granulocyte colony-stimulating factor, Leucogen tablets, Diyu Shengbai tablets to elevate white blood cell count, methylprednisolone sodium succinate for inflammation, and cefuroxime sodium for infection control.

**Outcomes:**

The patient was discharged after 11 days of clinical treatment.

**Lessons:**

Through the assessment of adverse drug reactions correlation and analysis of susceptibility factors, we recognized the importance of medication education and demonstrated that changes in clinical practice can save lives.

## 1. Introduction

Global data show that methimazole (MMI) and propylthiouracil (PTU)-induced agranulocytosis carries a high risk,^[1]^ are representative drugs for the treatment of hyperthyroidism. MMI is a thiourea drug (sulfonamide derivative) that reduces the production of thyroxine by inhibiting thyroxine synthesizing thyroid peroxidase, thereby effectively alleviating patient symptoms and restoring thyroid function. It has a longer half-life, and with clinical equivalent doses, MMI is 10 times more potent than PTU. The actual clinical effect is stronger than PTU, making it a first-line drug for clinical treatment of hyperthyroidism.^[2–4]^ MMI leads to a severe agranulocytosis with an incidence rate of 0.1%, which is a rare complication.^[5]^ The literature review indicates that MMI has the most significant effect on Th1 lymphocytes.^[6]^ This article reports a case of severe lymphocyte count reduction, febrile neutropenia, and White Blood Cell (WBC) count reduction in a patient with hyperthyroidism after using MMI tablets, and determines the type of adverse reaction. Combined with relevant literature, it analyzes the susceptibility factors of severe adverse reactions caused by antithyroid drugs (ATDs).

## 2. Case data

A 26-year-old Han Chinese female patient presented to hospital on October 8, 2024 due to palpitations and heat intolerance. Thyroid ultrasound examination revealed that the thyroid gland exhibited a plump shape with decreased and heterogeneous parenchymal echogenicity; color doppler flow imaging showed abundant blood flow signals within the thyroid; a lymph node approximately 4 × 3 × 5 mm in size was detected in the right cervical VI region, with a clearly visible cortico-medullary junction and thickened cortex; baseline labs were as follows: thyroid-stimulating hormone <0.005 µIU/L, free triiodothyronine 8.15 pmol/L, free thyroxine 35.80 pmol/L, WBC 5.85 × 10^9^/L. The patient was diagnosed with hyperthyroidism and initiated on MMI 15 mg once daily combined with adenine phosphate tablets 10 mg 3 times a day (tid) starting October 8, 2024. By October 18, labs revealed progressive cytopenia (WBC 4.16 × 10^9^/L, neutrophil [NEUT] 2.75 × 10^9^/L, lymphocyte [LYMPH] 0.82 × 10^9^/L). On November 6, she developed fever (39°C) and received antibiotics at the emergency department. On November 8, she was admitted to the in-patient hospital. Her past and personal histories were without significance. Vital signs were found as follows: temperature 37.1℃, Pulse 151 bpm, respiration 22/min, and blood pressure 115/87 mm Hg. Physical examination had revealed an enlarged tonsil (degree I) with purulence. admission diagnoses were made as follows (WBC 0.48 × 10^9^/L, NEUT 0.02 × 10^9^/L, LYMPH 0.39 × 10^9^/L, Red Blood Cell Count 4.44 × 10^12^/L, hemoglobin 112g/L): granulocytopenia with fever, thyroid storm, acute purulent tonsillitis, mild anemia. Integrated analysis of medication history suggested that the drug causing the severe decrease in lymphocyte count, WBC count, and granulocyte count was MMI tablets. MMI was discontinued and Human Granulocyte Colony-stimulating Factor 150 µg ih qd was given emergently to increase the WBC count. Leucogen tablets 20 mg po tid and Diyu Shengbai tablets 0.3 g po tid were prescribed to continuously increase the WBC count. Methylprednisolone sodium succinate 40 mg ivgtt qd and cefuroxime sodium 1.5 g ivgtt q8h were administered for anti-infection. On November 11, 2024, laboratory examination showed: WBC 0.91 × 10^9^/L, NEUT 0.04 × 10^9^/L, LYMPH 0.79 × 10^9^/L, and the current treatment plan was continued. November 14, 2024: auxiliary examination showed: WBC 3.19 × 10^9^/L, NEUT 0.38 × 10^9^/L, LYMPH 2.46 × 10^9^/L. November 18, 2024, laboratory examination showed: WBC 9.13 × 10^9^/L, NEUT 5.11 × 10^9^/L, LYMPH 3.29 × 10^9^/L, with WBC count, neutrophil count, and lymphocyte count rising to normal ranges. During this treatment cycle (10 days), the patient demonstrated high compliance and experienced no adverse drug reactions (ADRs). The patient was discharged on November 19, 2024. Based on patient data, I will analyze the drug causality assessment and susceptibility factors.

## 3. Standards for judging adverse drug reactions

### 3.1. New standards for judging adverse drug reactions

Pursuant to Chapter VIII, Supplementary Provisions of Measures for the Reporting and Monitoring of ADRs (Order No. 81 issued by the Ministry of Health of the People’s Republic of China: https://www.samr.gov.cn/cms_files/filemanager/samr/www/samrnew/samrgkml/nsjg/bgt/202106/W020211124522567235720.pdf), novel ADRs are defined through 2 distinct criteria: Any previously undocumented adverse reaction in the official drug labeling information. If there is a description in the package insert, but the nature, severity, consequences, or frequency of the adverse reaction is inconsistent with or more serious than the description in the package insert, it should be handled as a new adverse drug reaction.

### 3.2. Standards for judging severe adverse drug reactions

Referring to the US Department of Health and Human Services document *Common Terminology Criteria for Adverse Events Version 5.0* (https://dctd.cancer.gov/research/ctep-trials/for-sites/adverse-events/ctcae-v5-5x7.pdf), each adverse event is graded. Grading refers to the severity of the adverse event. Common terminology criteria for adverse events makes specific clinical descriptions of the degree of serious adverse event (grade 3–grade 5) based on the following basic criteria. Grade 3: severe or medically significant but not immediately life-threatening; hospitalization or prolongation of hospitalization indicated; disabling; limiting self care activities of daily living. Grade 4: life-threatening consequences; urgent intervention indicated. Grade 5: death related to adverse event (Table 1).

**Table 1:** Classification and grading of severe adverse events (SAEs).

CTCAE term	Grade 3	Grade 4	Grade 5	Case date	Adverse event
Lymphocyte count decrease	0.5–0.2 × 10^9^/L	<0.2 × 10^9^/L	–	0.39 × 10^9^/L	Grade 3
White blood cell decrease	1.0–2.0 × 10^9^/L	<1.0 × 10^9^/L	–	0.48 × 10^9^/L	Grade 4
Neutrophil count decrease	1.0–0.5 × 10^9^/L	<0.5 × 10^9^/L	–	0.02 × 10^9^/L	Grade 4

CTCAE = common terminology criteria for adverse events, SAEs = severe adverse events.

## 4. Drug association evaluation analysis

According to the causality assessment method of the National Adverse Drug Reaction Monitoring Center, the correlation of this adverse reaction event is *very likely*. The Naranjo’s scoring algorithm scores 7 points, with specific scores as follows: methimazole-induced granulocyte count and WBC count reduction have been reported (1 point); severe lymphocyte count reduction, WBC count reduction, and febrile neutropenia occurred after using MMI (2 points); ADRs were relieved after discontinuation of MMI (1 point); there was no cause other than the drug that caused the severe adverse reaction (2 points); through MMI medication time and auxiliary examination, it can be confirmed that this is an adverse reaction (1 point). In summary, the occurrence of severe adverse reactions is very likely related to MMI tablets (Table 2).

**Table 2:** Naranjo adverse drug reaction probability scale.^[7]^

Question	Yes	No	Do not know	Score
1. Are there previous conclusive reports on this reaction?	+1	0	0	1
2. Did the adverse event appear after the suspected drug was administered?	+2	−1	0	2
3. Did the adverse reaction improve when the drug was discontinued or a specific antagonist was administered?	+1	0	0	1
4. Did the adverse event reappear when the drug was readministered?	+2	−1	0	0
5. Are there alternative causes (other than the drug) that could on their own have caused the reaction?	−1	+2	0	2
6. Did the reaction reappear when a placebo was given?	−1	+1	0	0
7. Was the drug detected in blood (or other fluids) in concentrations known to be toxic?	+1	0	0	0
8. Was the reaction more severe when the dose was increased or less severe when the dose was decreased?	+1	0	0	0
9. Did the patient have a similar reaction to the same or similar drugs in any previous exposure?	+1	0	0	0
10. Was the adverse event confirmed by any objective evidence?	+1	0	0	1
Total score				**7**

## 5. Analysis of susceptibility factors for severe adverse drug reactions

### 5.1. Gene

In the Han Chinese population, human leukocyte antigen (HLA) HLA-B*27:05, HLA-B*38:02 and HLA-DRB1*08:03 were susceptibility HLA variants for ATD-induced agranulocytosis.^[8]^ MMI-induced severe granulocytopenia is associated with HLA-B*27:05, HLA-B*38:02, HLA-DRB1*08:03, rs2596487 and rs1811197 genes. Genome-wide association studies in Taiwanese and Hong Kong Chinese populations have shown that ATD-induced granulocytopenia is associated with HLA alleles HLA-B*38:02 and HLA-DRB1*08:03. In European Caucasians, ATD-induced granulocytopenia is associated with HLA-B*27:05 and other single nucleotide polymorphisms on chromosome.^[8,9]^ Japanese scholars have shown that HLA DRB1*08032 allele is closely related to the susceptibility of methimazole-induced granulocytopenia.^[10]^

### 5.2. Gender

Compared to men, women are more likely to develop granulocytopenia.^[11]^ Nakamura H statistically found that granulocytopenia occurred within 90 days after using ATDs, with an average age of 43.4 ± 15.2 years and a male-to-female ratio of 1:6.3.^[12]^ The number of female patients increased with age. Female estrogen and X chromosome inactivation and microchimerism can change the immune system. During pregnancy, menstrual cycles, and menopause, estrogen fluctuations affect disease changes. Estrogen receptor ESR2 polymorphism is common in Graves’ disease (GD), which also indicates the importance of estrogen in its pathophysiology.^[13]^

### 5.3. Age

ATD-induced granulocytopenia is a rare and life-threatening adverse reaction, with a mortality rate of up to 21.5% for granulocytopenia (MMI-induced agranulocytosis, MMI-AGRAN). It is considered that ATD-induced granulocytopenia may be related to age.^[12,14–16]^ Younger children and elderly patients may have a higher risk of developing granulocytopenia.^[16,17]^ It has been reported that younger children or children with lower neutrophil counts may face a higher risk of neutropenia.^[17]^ In addition, with aging, organ function declines, drug metabolism is impaired, the immune system is weakened, sensitivity to drugs increases, tolerance and regulation abilities decrease, and drug accumulation in the body leads to adverse drug interactions. The risk of MMI-AGRAN increases over 40 years of age, and the mortality rate of MMI-AGRAN increases over 60 years of age. Elderly people are more likely to develop granulocytopenia MMI-AGRAN.^[16]^

### 5.4. Dosage

MMI is the most commonly used drug for the treatment of hyperthyroidism. Improper selection of the initial dose will affect the efficacy. Too high a dose will cause various adverse reactions, while an insufficient dose will make it difficult to achieve the expected effect.^[18]^ Older patient age and higher drug doses are risk factors for cholestatic injury.^[19]^ It has been reported that the incidence of granulocytopenia with low-dose (≤15 mg/d) MMI regimens is much lower than with high-dose regimens.^[20]^ According to the *Guidelines for Rational Drug Use at the Primary Level for Hyperthyroidism*,^[21]^ The initial dose is 20 to 40 mg/d, which is divided into 1 to 2 doses, and the condition improves in 2 to 6 weeks. Then the initial dose can be gradually reduced to a maintenance dose. In conservative treatment of hyperthyroidism, the conservative course of treatment of hyperthyroidism is usually 6 to 24 months. MMI-AGRAN may occur in the first 3 months of treatment, so close monitoring is especially needed during this period 9. After completing the standard course of MMI treatment, if thyroid function has reached a normal state and no adverse reactions have occurred, a long-term continuous small dose of 2.5 to 5 mg of MMI can be used daily to prevent recurrent hyperthyroidism or maintain normal thyroid function.^[22]^

### 5.5. Dosage form

Oral ATD plays an important role in the treatment of hyperthyroidism in clinical practice.^[23]^ MMI is the most commonly used drug for the treatment of hyperthyroidism, with good efficacy and few adverse reactions.^[18]^ The drug instructions state that MMI tablets can be rapidly absorbed in the gastrointestinal tract after oral administration, with an absorption rate of about 70% to 80%, distributed throughout the body, concentrated in the thyroid gland, but it does not bind to proteins in the blood The T1/2 of MMI is about 3 hours, and the biological effect can last for a considerable period. MMI ointment is a new type of transdermal absorption preparation, which can directly enter the circulatory system through the capillaries of the subcutaneous tissue, promoting the accumulation of active ingredients in the thyroid gland. Compared with oral preparations, MMI ointment has a lower concentration in serum and a higher concentration in thyroid tissue, and its clinical effect is similar to that of oral tablets. Choosing MMI ointment might not only improve the efficacy but also reduce the amount of drug entering the circulatory system, thereby reducing the occurrence of systemic adverse reactions.^[24]^

### 5.6. Interactions

The interaction between MMI and other drugs is more complicated. Clinicians should be cautious when using combined drugs and need to monitor the patient’s thyroid function during medication to ensure the safety and effectiveness of the treatment. According to literature reports, both ATDs and phenothiazines can cause granulocytopenia, and the combined use of the 2 drugs can increase the risk of granulocytopenia.^[25]^ In addition, changes in thyroid hormone concentration can change the degree of warfarin anticoagulation.^[26]^

### 5.7. Medication education

Usually, medication education for patients when starting to use ATDs can prevent death caused by ATD drug-induced granulocytopenia.^[5]^ According to literature, more than 80% of patients with granulocytopenia may experience symptoms of fever and sore throat, and more than 60% of patients using ATDs do not know the symptoms of granulocytopenia.^[4,5,8]^ The lower the granulocyte count, the longer the recovery time. Therefore, medication education for patients is crucial for preventing death and improving prognosis. MMI is a sulfonamide derivative and the first choice for treating hyperthyroidism.^[2]^ According to the *Guidelines for the Diagnosis and Management of Hyperthyroidism and other causes of Thyrotoxicosis in China*^[4]^: It is recommended to take it at the same time every day after meals, and do not arbitrarily increase or decrease the dose and frequency of medication. According to the MMI tablet instructions, common adverse reactions include rash, joint pain, muscle pain, nausea, upper abdominal pain, headache, drowsiness, and edema; severe adverse reactions include pancreatitis, granulocytopenia, aplastic anemia, leukopenia, eosinophilia, thrombocytopenia, hypoprothrombinemia, liver necrosis, insulin autoimmune syndrome, nephritis, lymphadenopathy, loss of taste, and neuritis. Patients should be instructed: If symptoms such as fever, sore throat, nausea, vomiting, rash, or yellow urine occur, seek medical attention promptly. In the first 3 months of treatment, monitor blood routine once a week and liver function once a month. For maintenance treatment, it is recommended to monitor blood routine once a month and monitor thyroid function regularly. During medication, avoid taking iodine preparations and high-iodine foods. If using other drugs, you need to inform relevant medical personnel to avoid drug interactions that reduce efficacy and increase toxicity.^[22]^

## 6. Discussion

The patient was a 26-year-old Han Chinese woman who used MMI tablets 15 mg for 1 month to treat hyperthyroidism while using adenine phosphate tablets (according to the drug’s pharmacological action described in the package insert, adenosine phosphate (https://db.yaozh.com/instruct/43250.html) is a leukocyte-increasing agent, and vitamin B_4_ is a nucleic acid component involved in RNA and DNA synthesis, which promotes leukocyte proliferation when leukopenia occurs. It is used to prevent and treat leukopenia and acute granulocytopenia caused by various reasons to prevent granulocytopenia. One month later, the patient developed chills, fever, sore throat, and headache and was unaware of the symptoms of granulocytopenia, indicating that medication education is crucial for patients with severe adverse reactions and prevention of death. Clinicians should provide proper medication education to patients, advising regular blood monitoring. Follow-up schedules should be communicated and reminders sent via phone calls, short message service, public accounts, or email. Furthermore, a combination of online and offline approaches should be used to promote medication safety: online through web platforms, offline using standardized, visual warning cards indicating symptoms such as fever, sore throat, rash, dyspnea, dark urine, nausea, and abdominal pain requiring immediate drug discontinuation and emergency contact information.

After reviewing PubMed, Foreign Medical Literature Retrieval Service, Web of Science, China National Knowledge Infrastructure, Wanfang Data, and VIP Knowledge Resource Platform, literature was found on methimazole-induced agranulocytosis and leukopenia case reports.^[27,28]^ However, no case reports of methimazole-induced severe lymphocytopenia were found. This severe adverse reaction report has certain novelty. We anticipate that this case report will contribute valuable insights to enhance clinical diagnostic safety and therapeutic decision-making in similar cases.

Novel severe ADRs emerged following 1-month methimazole treatment in the patient. Analyzing the reasons for the occurrence of ADRs, genes and gender are susceptibility factors, and a higher dose of MMI tablets is also one of the reasons for the occurrence of serious adverse reactions. Long-term continuous use of a small daily dose of 2.5 to 5 mg of MMI can prevent recurrent hyperthyroidism or maintain normal thyroid function.^[22]^ In addition, dosage form might be also one of the susceptibility factors for serious adverse reactions. It is considered to try MMI ointment after relief of mild neutropenia, lymphocyte count reduction, leukopenia because MMI ointment reduce the amount of drug entering the circulatory system, thereby reducing the incidence of serious adverse reactions.

China’s rare reported case of severe lymphocytopenia induced by MMI: This case systematically demonstrates the causal relationship between MMI administration and progressive lymphocytopenia via meticulous chronological evaluation. However, due to 1 case report, the study may not fully reflect all potential ADRs, there are certain limitations to the case report.

## 7. Summary

Severe adverse reactions includes 7 susceptibility factors, which are genes, gender, age, drug dosage form, higher doses, interactions, and medication education. Clinical attention should be paid to medication education. It is worth noting that changes in clinical practice may save lives.

## Acknowledgments

The authors would like to thank the editors and reviewers for the helpful comments.

## Author contributions

**Writing – original draft:** Yi Li, Bo Tan.

**Writing – review & editing:** Yi Li, Bo Tan, Liutao Zheng.
